# Artificial intelligence assisted acute patient journey

**DOI:** 10.3389/frai.2022.962165

**Published:** 2022-10-04

**Authors:** Talha Nazir, Muhammad Mushhood Ur Rehman, Muhammad Roshan Asghar, Junaid S. Kalia

**Affiliations:** ^1^Research Fellow, NeuroCare.AI Neuroscience Academy, Dallas, TX, United States; ^2^NeuroCare.AI, Dallas, TX, United States; ^3^Neurologypocketbook.com, Dallas, TX, United States

**Keywords:** artificial intelligence, acute patient journey, electronic-triage, health IoT, Automated EMR summary, AI-based clinical decision support system

## Abstract

Artificial intelligence is taking the world by storm and soon will be aiding patients in their journey at the hospital. The trials and tribulations of the healthcare system during the COVID-19 pandemic have set the stage for shifting healthcare from a physical to a cyber-physical space. A physician can now remotely monitor a patient, admitting them only if they meet certain thresholds, thereby reducing the total number of admissions at the hospital. Coordination, communication, and resource management have been core issues for any industry. However, it is most accurate in healthcare. Both systems and providers are exhausted under the burden of increasing data and complexity of care delivery, increasing costs, and financial burden. Simultaneously, there is a digital transformation of healthcare in the making. This transformation provides an opportunity to create systems of care that are artificial intelligence-enabled. Healthcare resources can be utilized more justly. The wastage of financial and intellectual resources in an overcrowded healthcare system can be avoided by implementing IoT, telehealth, and AI/ML-based algorithms. It is imperative to consider the design principles of the patient's journey while simultaneously prioritizing a better user experience to alleviate physician concerns. This paper discusses the entire blueprint of the AI/ML-assisted patient journey and its impact on healthcare provision.

## Introduction

Artificial intelligence is being used in the industry to leverage data for logistics. Previously, decisions were made at a human level to shelf individual products in a supermarket. Now we see that data enhances human decisions to find the right product and make seasonal recommendations. Similarly, AI will be helping in the complete journey of patients in terms of pre-hospital alert and in-hospital stay, and eventually, creating a pathway for post-hospital care. The healthcare industry is shifting its focus from decreasing readmissions to reducing admissions (Kang et al., 2020). Telehealth, health IoT, and other medical devices are being introduced every day. The FDA has recently published a general framework to streamline the integration of medical devices into the healthcare system (FDA, 2021).

Almost 25% of the US healthcare budget is being wasted due to multiple factors such as lack of coordination, over-treatment or low-value care, complex administrative procedures, and failure to provide care. Also, the healthcare system is getting crowded due to fewer human resources available (Shrank et al., 2019). We have observed the collapse of the healthcare system during the COVID-19 pandemic, and many patients with chronic diseases were left without a physician's consultation. We can improve healthcare provision by leveraging IoT, telehealth, and AI/ML-based algorithms with clinical decision support systems. AI/ML-based applications help apply the 4p model of healthcare (predictive, preventive, personalized, and participatory) (Briganti and Olivier, 2020).

**Figure 1:**
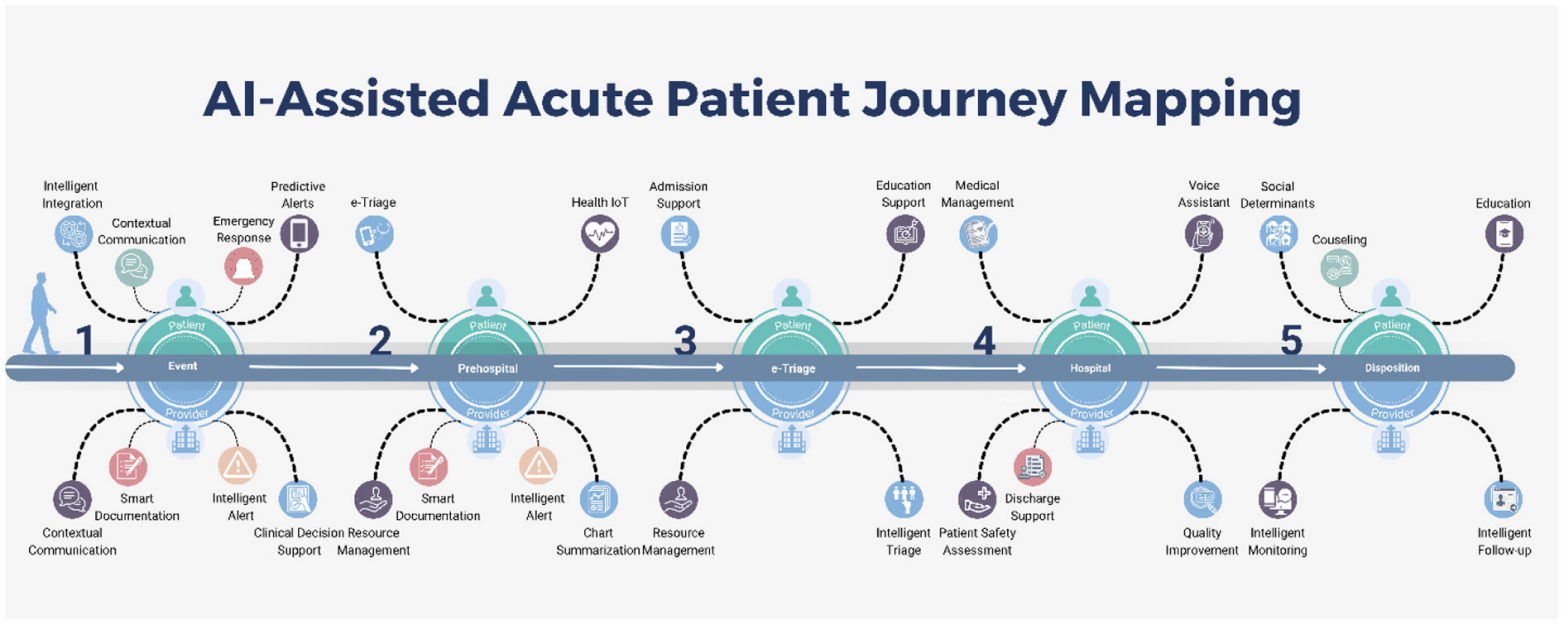
Al-Assisted Acute Patient Journey Mapping.

Unfortunately, the current available solutions are isolated, and the true power of digital generation to create value in medicine comes from an ecosystem approach. Acute care is the most expensive portion of the United States healthcare system. This paper presents a narrative review of artificial intelligence technologies that can provide value to the community and insight to patients for better healthcare management. It aims to explain how a network of small digital solutions working together in coherence can impact healthcare provision throughout the patient's journey (Figure 1). Furthermore, it provides a broader picture of what the digitization of healthcare means for the future.

## Discussion

AI/ML has made a significant contribution to a patient's journey from their homes to being released from the hospital. It can aid in gathering data from wearables and earlier health records and, in the end, ingeniously compile a patient's medical history. Furthermore, E-triage and smart clinical decision support systems can help in the allocation of resources more effectively, which in turn will aid in reducing healthcare spending without compromising the standard of care.

Digital tools are now ubiquitous in healthcare, whether in wearable devices or smart monitors in emergency rooms. While digital tools can help patients in a variety of ways, we have focused on the journey of acute patients and highlighted the impact of AI/ML tools on care delivery.

### Pre-hospital

One of the critical issues is the identification of patients that require a higher level of care in the pre-hospital settings. There have been many systems based on statistical modeling to generate automated emergency alerts and predict the necessary levels of care, but they lack continuous learning and contextual reinforcement.

#### Automated emergency response/automated alert system

Automated alert systems may play an essential role in individualized emergency and disaster management. We have limited systems for acute emergencies with connected EMS systems (Pulsara, 2014). These systems need to be expanded, and more intelligent systems that link to wearable devices need to be developed.

The interest in applications such as fall and lost detection has risen over the past few years. Wang and the team reviewed the fall detection devices and concluded that these devices focus on offline analytics instead of automated healthcare monitoring. In addition, Apple has already implemented an emergency alert system and an automated fall detection system in the Apple Watch (Apple., 2022). We need to increase the sensitivity and specificity of fall detection and integrate it into our existing healthcare system to generate accurate and automated alerts. Furthermore, we can replicate the same process for other problems (Wang et al., 2020).

#### Healthcare data

We live in a digital world, and digital health as a reality relies on data. Machine learning (ML) modules require large amounts of high-quality data as training data to produce “ground truth.” We need to make high-quality real-time data collection a priority. This also creates a double-edged sword, opening the door to privacy and security concerns. Governance and management of data are central to creating secure data warehouses that provide access with differential privacy. Newly developed ML models like federated learning and swarm learning have brought new methods of decentralized learning. These models of decentralized learning will be crucial in healthcare as they provide privacy in learning, minimizing the need for a centralized data warehouse, and are more resourceful as they can push learning to the edge, decreasing cost and latency (Herresthal et al., 2021).

In these times of information explosion, it is difficult for healthcare workers to sift through a large amount of data to find pertinent information. AI-based applications are crucial to separating signal from noise. With modern data visualization techniques, these results, like pertinent labs, and imaging, can be presented in an interactive format. Physicians can rapidly identify and act on collected data with contextual information to make better-informed decisions (Stanfill and Marc, 2019). Additionally, by analyzing general population behavior and discovering new research avenues (Karan et al., 2022) through user-generated content (Saura et al., 2020) on social media, we can effectively fill any gaps in healthcare provision. This is the need of time to revolutionize the patient care paradigms and establish best practices to allocate financial resources, as the healthcare industry faces a heavy financial burden (Cai et al., 2020).

#### Wearable devices and eHealth integration

**Figure 2:**
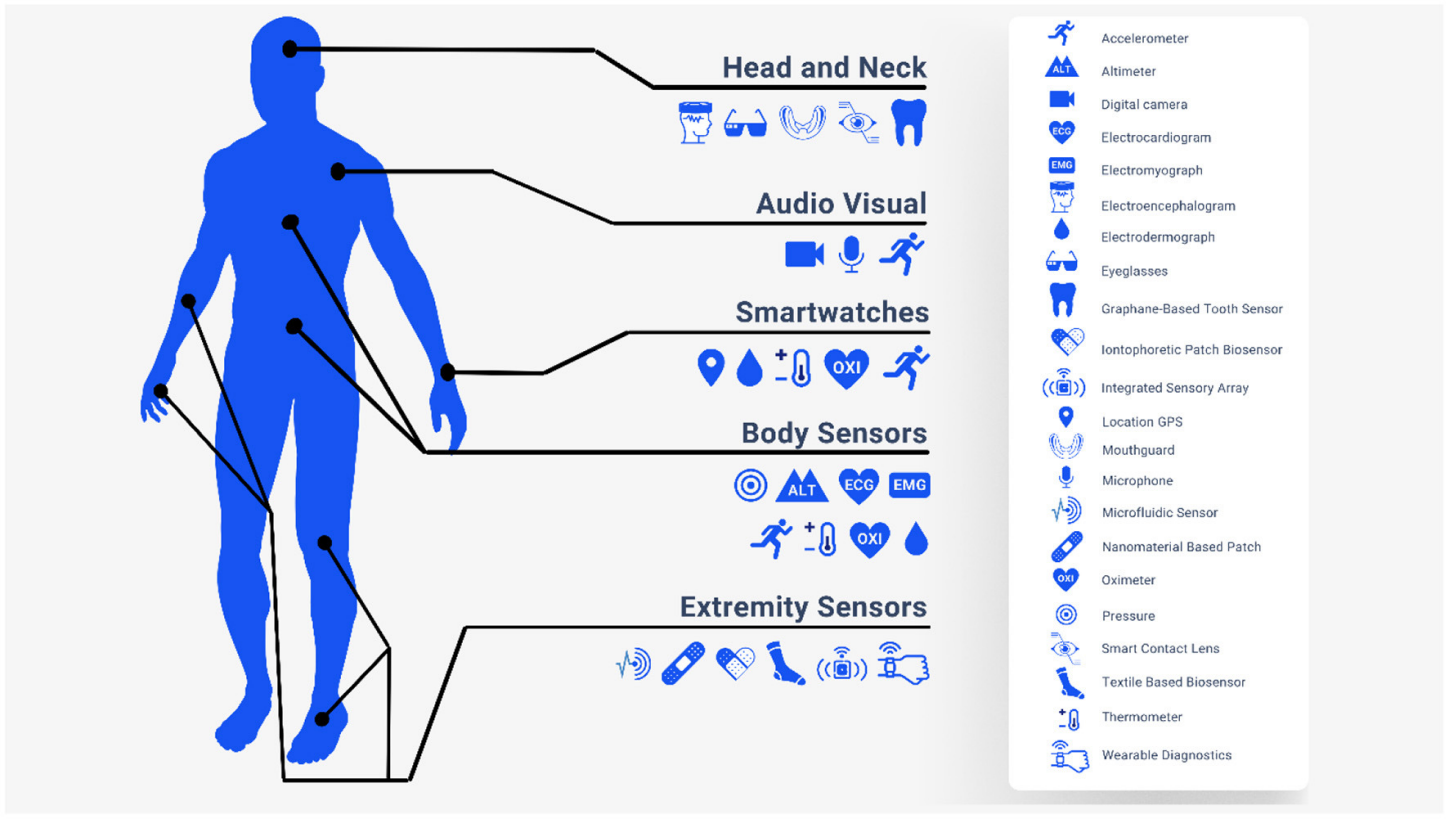
Health Internet of Things (IoT).

The adoption of wearables is increasing each year. More importantly, these are increasingly being integrated into healthcare. Important patient data like blood pressure, pulse, oxygen saturation, temperature, ambulatory ECG, seizure, and stroke alerts (Figure 2) collected from various devices is being integrated using a software development kit (SDK) by Google Fit™ and Apple HealthKit™ (Henriksen et al., 2018). As an example of wearables in healthcare, Embrace 2 detects seizures and notifies users (Embrace, 2018). This provides an opportunity for a collaborative and participatory healthcare environment between the patient and provider. When linked to emergency response services, these systems can significantly decrease latency and cost of care. More importantly, it will enable clinical decision support tools for diagnosis and management (Dinh-Le et al., 2019).

Rather than functioning as a standalone entity, mobile healthcare applications should be integrated into the existing healthcare system. mHealth applications can assist us in deciphering data from wearables and other smart devices. The lack of a standard framework for innovators to work on these avenues is the main barrier to innovation. Labrique and the team proposed a common framework for health-based that addressed all the essential components of the healthcare system, starting with communication, data collection and going all the way to financial transactions and incentives (Labrique et al., 2013). In 2019, the FDA also announced the pre-certification program, establishing a framework for regulating digital health goods (FDA, 2019). The development of a common framework will incentivize the process of innovation in this field.

#### Electronic-triage and severity index

The capabilities and availability of an electronic triage system (ETS) are improving. Currently, it is only available for a limited number of diseases and in a limited number of facilities. The core problem is the accuracy of determining disease severity. The Emergency Severity Index (ESI) is a commonly used severity indexing system in the USA. However, most patients fall into level 3 on the ESI. Level 3 is a middle ground between high severity requiring extensive resources and low severity with a decreased resource requirement. This, in essence, does not guide physicians in terms of resource allocation. Levin et al. (2018) applied a machine learning model to improve the accuracy of level 3 on ESI. They applied the random forest model to the ESI level 3 patient triage data (65% of 172,726 ED visits). ETS tagged almost 22.9% as level 1, compared to 16.9% by ESI. Patients who were up-triaged to level 2 or 1 by ETS were prone to a critical outcome by almost five times and two times more likely to get hospitalized, and similarly decreased the risk of a critical event in patients who were down-triaged to level 4 or 5. Also, the study showed that the detection rate of secondary clinical outcomes was similar to or better than ESI (Levin et al., 2018).

Similarly, Kang et al. (2020) developed an AI algorithm to predict the need for critical care and compared it to existing triage systems. The combination of the AI algorithm and ESI outdid all other scoring systems (Kang et al., 2020). Integration of AI/ML-based tools into existing EMS can help streamline the care of patients in the ED.

### Resource allocation

Machine learning can be used for appropriate resource allocation to alleviate the overcrowding of ERs. Predicting the length of stay in the ER can help streamline the workflow and prevent resource waste. Yousaf and the team have developed a novel algorithm to decrease the length of stay in ED. By combining a chaotic genetic algorithm and Adaboot (meta-learning), they managed to reduce the length of stay from 5.47 to 4.75 h in public testing at the emergency department of the Recoleta Tolentino Neves Hospital, Brazil. By actively monitoring vitals and labs remotely during the transfer and on-site, AI/ML algorithms can reduce ED resource usage by providing clinical decision support and appropriate allocation of resources (Yousefi et al., 2018).

#### Clinical decision support systems

AI-based clinical decision support system (AI-CDSS) is another avenue that needs to be explored. The poor integration of the existing clinical decision support system can lead to alarm fatigue and physician burnout. However, there are examples to follow in AI-CDSS implementation to improve patient care and provider satisfaction. The Canadian Association of Radiologists has explained in their recent paper that AI-based analysis of imaging will be more sophisticated and easier to integrate into our workflow. The diagnostic accuracy of lung nodules and congenital cataracts has been proven to be comparable to that of a trained physician (Stanfill and Marc, 2019).

Duke University Hospital has implemented a sepsis watch tool to detect early signs of sepsis in patients. Despite the lack of previous experience with integrating algorithms based on AI/ML, they could incorporate the tool into their workflow. An algorithm was trained to create an alert almost 12 h before the presentation. Early analysis showed that the median detection time was 5 h before the patient's deterioration, providing ample time for the physician to confirm the diagnosis and intervene (Sendak et al., 2020).

#### Clinical monitoring

Medical monitoring devices are based on the threshold alarming principle and do not contain analytical functions. AI/ML-based applications can help analyze large amounts of data and detect subtle clinical anomalies that humans may overlook (Rush et al., 2019). Remote clinical monitoring is vital in monitoring chronic diseases. COVID-19 pandemic has dramatically pushed the healthcare industry toward telehealth and remote monitoring devices. The remote monitoring of blood glucose levels, atrial fibrillation, epilepsy, BP, pulse, temperature, and oxygen saturation can significantly help physicians intervene timely. Also, patients per day visiting can be reduced by resolving minor problems with the help of telehealth and remote monitoring.

#### Clinical documentation

Documenting clinical journeys with contextual information is helpful for patient pathology accounts and is critical to maintaining quality standards. There is a significant burden for patients and allied health professionals (clinical coordinators, nurse educators, EMS responders) in terms of time to ensure proper time-stamped documentation. Several technologies have recently been introduced to create an ambient intelligence environment. These technologies can improve the quality of documentation while simultaneously reducing the burden on clinicians. (1) Automated Voice Transcription, (2) Digital Voice Assistant for providers, and (3) Voice assistant for patients. However, these technologies need to be incorporated and interoperable with deep integration at a system level to achieve accurate ambient intelligence (Microsoft, 2021).

Accurate clinical documentation leads to accurate medical coding, which is essential for (1) reimbursement, (2) quality improvement, and (3) future resource allocation planning. Implementing a common framework is a crucial requirement to allow the interoperability of AI/ML algorithms in the healthcare industry. This standardization and common framework will allow us to have resource planning at the level of a state, country, or even continents compared to county and hospital systems (Stanfill and Marc, 2019).

#### Automated EMR summary

The current EHR applications are hindering the provision of healthcare rather than helping it. The process of finding relevant information is usually manual. EHR systems can be improved by introducing customized EHR systems, using open-source software and customizing it, and incorporating AI/ML-driven applications. Some famous companies such as Epic, Allscripts, Cerner, and Athena are introducing AI/ML-based EHR tools and decision support systems to tackle the problem of data explosion. Many other startups and big names, such as Amazon Web Services and Google, have also introduced cutting-edge AI/ML-based tools (Davenport et al., 2018).

Rajkumar and the team published an article discussing the role of deep learning in developing predictive models and recommending that the Fast Healthcare Interoperability Resource (FHIR) be utilized. They have validated their hypothesis by using medical records from two hospitals. Results showed that the deep learning predictive model had outperformed the conventional prediction models in predicting mortality, prolonged length of stay, readmission, and discharge diagnosis. Deep learning predictive models can eliminate many variables that are the main hurdle in conventional predictive models. In addition to presenting relevant charts, history, and labs, to the physicians and paramedical staff, AI/ML integration can also help develop future learning aids (Rajkomar et al., 2018).

### Disposition and continuity of care

As mentioned above, AI can be crucial for judicious, value-based resource allocation. However, it can be instrumental in automating patient continuity of care. One of the critical issues in mapping the patient journey is quickly moving the patient toward rehabilitation and a home environment. Patients with continuous monitoring will give us the data to make decisions over a longitudinal period compared to brief clinical visits by providers, even in an inpatient setting.

For better health outcomes, it is crucial to avoid unnecessary hospital stays and timely discharge of patients. AI/ML-based models can predict the patients who should be discharged based on their medical records and detect the barriers to discharge (Safavi et al., 2019). Mitigating those barriers can significantly reduce the anxiety of extended stays of patients on one hand while optimizing resource allocation and healthcare workers' efficiency on the other. As shown by a TEND Model study (van Walraven and Forster, 2018), AI models can predict the number of patients discharged per day. As data becomes more accessible, it will reduce costs and avoid repeating labs. Providing progressive summaries should be an integral part of the patient journey, and AI can aid providers. It can help transfer medical records along with patients without any loss of information.

As we noted above, we can use wearables not only to detect events but also to improve follow-up visitation frequency both in-person and *via* telehealth. Bian et al. (2020) conducted research at Peking Union Medical College Hospital. They concluded that AI-assisted follow-up of individual patients is comparable to manual follow-up by phone calls but in a much lesser time, i.e., 0 h compared to 9.3 h per 100 patients. Such interventions can be made sooner *via* notification. Physicians can provide care by telepresence or in person, depending on the patient's situation, rather than assigning arbitrary 2, 4, 6-week visits (Bian et al., 2020).

### Health education

Education of the patient and the patient's family is essential to recovery, and many chronic conditions require a higher level of care. Relevant, just-in-time, and mixed media education can significantly improve patients' motivation, adherence, and compliance with medication and rehab therapy. Also, given the electronic nature and reproducibility, the patient can receive education about specific conditions, further increasing the chance of compliance.

Point-of-care education enhanced by AI/ML. Monitoring from various devices can be integrated into one application that can show notifications to educate patients according to their physical needs and current medical status. This kind of tailored education can improve the health outcomes of patients significantly.

## Challenges and limitations

Though the advent of digital health tools will improve healthcare, it comes with challenges such as the reliability and validity of mHealth devices, access of the third party to patients' data, and lack of patient data management. Similarly, continuous monitoring might increase stress and raise concerns about patients' health (Volpato et al., 2021). Explainability is another critical challenge. Occasionally, the involved doctor may find it difficult or impossible to explain the algorithms responsible for the diagnosis to the patient (Davenport and Kalakota, 2019). Patients' privacy is another big concern as we propose more integration of healthcare data to produce wisdom through AI. More integrated systems are more vulnerable to cyberattacks and data theft.

There are substantial ethical challenges in AI implementation in healthcare. Accessibility and inequality have already profoundly impacted healthcare. AI can be expensive, and large portions of humanity may not have access to these tools because companies are charging premium prices for their services. Hence, it has the potential to deepen information inequality as well.

The bidirectional flow of information between healthcare providers and patients is crucial for continuously monitoring patients at risk for recurrent episodes of life-threatening disease. Therefore, it is critical to have a certain level of consistency among digital health tools in predicting the severity of symptoms in out-of-hospital patients. The significant challenges that hinder the implementation of these tools range from their reliability to their integration into existing health systems.

## Conclusion

Digital healthcare, like digital banking, will be the future norm. We need to use a design process that enables intelligent communication, coordination, and resource management during the whole journey of acute patients instead of introducing isolated solutions. By integrating smart solutions into the healthcare system as a whole, we can save a tremendous amount of money and enhance healthcare delivery. This type of solution will not be limited to acute settings but can be implemented in outpatient, mobile clinics, and other touchpoints in a patient's journey. We will continue to see further convergence of technologies, enabling new experiences for both patients and providers.

## Author contributions

TN had worked on literature research and writing the manuscript along with MM. MA was tasked on researching the literature and keep us updated regarding new modalities and publications and later on worked on limitations section. JK was our mentor who had supervised and helped us in formulating the table of content, editing, writing certain sections of manuscript etc. All authors contributed to the article and approved the submitted version.

## Funding

JK personally paid for this research article.

## Conflict of interest

Authors TN, MM and MA were employed by company NeuroCare.AI Neuroscience Academy. Author JK was employed by company NeuroCare.AI and Neurologypocketbook.com.

## Publisher's note

All claims expressed in this article are solely those of the authors and do not necessarily represent those of their affiliated organizations, or those of the publisher, the editors and the reviewers. Any product that may be evaluated in this article, or claim that may be made by its manufacturer, is not guaranteed or endorsed by the publisher.
